# Supplementary material for: Splice-Junction-Based Mapping of Alternative Isoforms in the Human Proteome
Source: Cell Rep. Author manuscript; Available in PMC 2020 Jan 15. (PMC6961840; doi:10.1016/j.celrep.2019.11.026)
Supplement: 3 [file NIHMS1546469-supplement-3.zip › DF2/PXD009737/TestisGluC-163-Q6UN15-VIPVTSTTNKITVQQGRTGNSEKE.pdf]

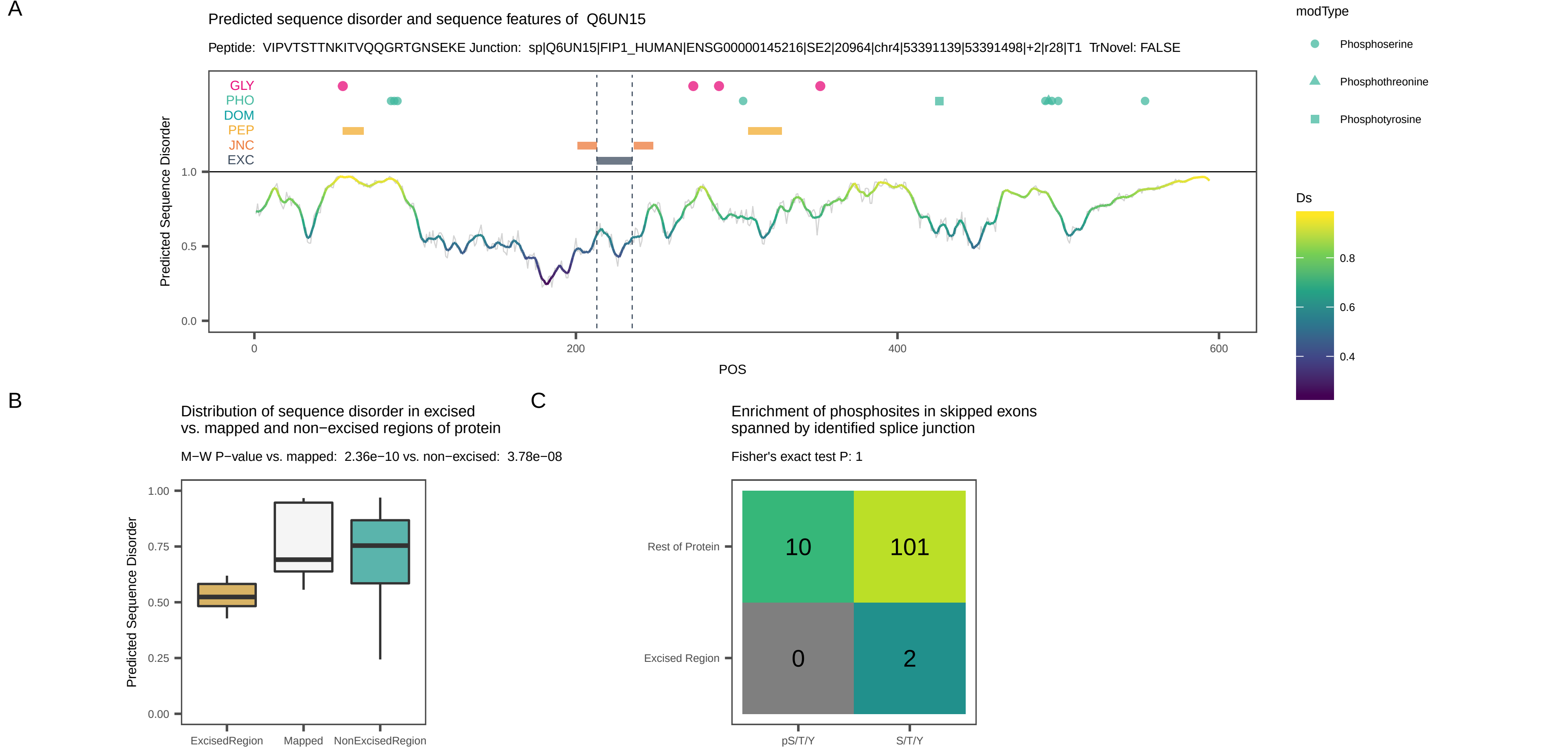

A
Predicted sequence disorder and sequence features of Q6UN15
Peptide: VIPVTSTTNKITVQQGRTGNSEKE Junction: sp|Q6UN15|FIP1_HUMAN|ENSG00000145216|SE2|20964|chr4|53391139|53391498|+2|r28|T1 TrNovel: FALSE
GLY
PHO
DOM
PEP
JNC
EXC
Predicted Sequence Disorder
1.0
0.5
0.0
0
200
400
600
POS
modType
Phosphoserine
Phosphothreonine
Phosphotyrosine
Ds
0.8
0.6
0.4
B
Distribution of sequence disorder in excised vs. mapped and non−excised regions of protein
M−W P−value vs. mapped: 2.36e−10 vs. non−excised: 3.78e−08
Predicted Sequence Disorder
1.00
0.75
0.50
0.25
0.00
ExcisedRegion
Mapped
NonExcisedRegion
C
Enrichment of phosphosites in skipped exons spanned by identified splice junction
Fisher's exact test P: 1
Rest of Protein
Excised Region
10
101
0
2
pS/T/Y
S/T/Y
